# Prevalence and Diversity among *Anaplasma phagocytophilum* Strains Originating from *Ixodes ricinus* Ticks from Northwest Norway

**DOI:** 10.1155/2014/824897

**Published:** 2014-08-24

**Authors:** Ann-Kristin Tveten

**Affiliations:** Faculty of Life Sciences, Aalesund University College, 6025 Aalesund, Norway

## Abstract

The tick-borne pathogen *Anaplasma phagocytophilum* causes great concern for livestock farmers. Tick-borne fever is a widespread disease in Norway, and antibodies have been produced amongst sheep, roe deer, red deer, and moose. The main vector *Ixodes ricinus* is found along the Norwegian coastline as far north as the Arctic Circle. A total number of 1804 *I. ricinus* ticks were collected and the prevalence of the pathogen was determined by species-specific qPCR. The overall infection rate varied from 2.83% to 3.32%, but there were no significant differences (*p* = 0.01) in the overall infection rate in 2010, 2011, or 2012. A multilocus sequencing analysis was performed to further characterise the isolates. The genotyping of 27 strains resulted in classification into 19 different sequences types (ST), none of which was found in the MLST database. The nucleotide diversity was for every locus <0.01, and the number of SNPs was between 1 and 2.8 per 100 bp. The majority of SNPs were synonymous. A goeBURST analysis demonstrated that the strains from northwest Norway cluster together with other Norwegian strains in the MLST database and the strains that are included in this study constitute clonal complexes (CC) 9, 10, and 11 in addition to the singleton.

## 1. Introduction

*Anaplasma phagocytophilum,* formerly* Ehrlichia phagocytophila*, is a vector-borne pathogen known to cause tick-borne fever (TBF) in ruminants and human granulocytic anaplasmosis (HGA) [1].* A. phagocytophilum* of the order Rickettsiales is a Gram-negative bacterium that invades neutrophils [1, 2].* Ixodes* ticks act as natural reservoirs for the bacterium. Uninfected ticks can acquire the pathogen while feeding on an infected mammal and can transmit the pathogen to mammals during a blood meal [3]. In Norway,* Ixodes ricinus* ticks are the main vector for* A. phagocytophilum*, and although HGA is not a common disease in Norway [4–6],* A. phagocytophilum* antibodies have been detected in sheep, roe deer, red deer, and moose [7]. The clinical symptoms include fever, leucopenia, and thrombocytopenia [8]. During an* A. phagocytophilum* infection, the 44 kDa major surface protein (msp2) plays an important role in adhesion to the surface receptors of neutrophils [9].* A. phagocytophilum* colonises within the invaded cells and interferes with the normal cellular function, thus affecting the normal regulation of the immune response [10]. A deprived immune response enables secondary infections to thrive and cause severe illness and even death [3].

The complete* A. phagocytophilum* genome sequence has been assembled, and like that of other* Anaplasma* spp. and* Ehrlichia* spp., it contains one circular chromosome of approximately 1.2–1.5 × 10^6^ bp. Some of the characterised genes are housekeeping loci, but the majority of the genes code for hypothetical or uncharacterised proteins [11]. The molecular characteristics of* A. phagocytophilum* include sequence studies of the 16S rRNA region, the surface membrane proteins* mps4* and* mps2*, the* groEL* locus, the* ankA* locus [12–14], or a combination of these [15]. The 16S rRNA genetic region is highly conserved and has been previously applied to characterise strains [16, 17]. The characterisation of strains by 16S rRNA sequences has identified 15 variants, some geographically located to either specific countries or continents and some distributed worldwide [12]. Several 16S rRNA variants of the bacterium have been found in mammals and ticks in Europe. Vector-host interaction, genotypes, geographical distribution, and pathogenesis are recognised as biological and ecological differences between the identified genetic variants of* A. phagocytophilum* [18]. Some studies indicate that a host could be infected with several different genetic variants at the same time and that these variants behave differently and affect each other inside the host [19]. Sheep that have been infected with different genotypes have presented different clinical manifestations, and different genetic variants can be detected at different times [5, 18, 20, 21]. In addition to the 16S rRNA locus, the major surface proteins (*msp2* and* mps4*) and the* ankA* locus have been targeted in studies of the genetic variation between* A. phagocytophilum* strains [13, 22, 23]. Antigen variations within the major surface proteins are assumed to play a key role in enabling* A. phagocytophilum* to persist in mammals [18]. Multilocus sequence typing (MLST) is a highly discriminatory genotyping method that enables a more detailed delineation. The method can provide genetic information on strains from different geographic origins and identify nucleotide diversity, polymorphism rates, and genetic distances. The MLST network enables laboratories to compare their sequence strains with those from other laboratories [24–26]. The multilocus sequence typing (MLST) scheme for* A. phagocytophilum* is curated by Von Loewenich et al. through the MLST network at http://pubmlst.org/aphagocytophilum/. This scheme is based on the nested amplification and sequencing of the seven housekeeping loci* atpA*,* dnaN*,* fumC*,* glyA*,* mdh*,* pheS*, and* sucA* and is designed to provide a detailed description of* A. phagocytophilum* strains to describe strain diversity and evolutionary development.

The aim of this study was to determine the prevalence of* A. phagocytophilum* in* I. ricinus* ticks from northwest Norway and study the genetic variation between the strains via MLST.* I. ricinus* ticks are the main vector for obtaining and transmitting the pathogen in this region. The genetic characterisation of strains could provide new knowledge of the strain diversity of* A. phagocytophilum* in this region.

## 2. Materials and Methods

A total of 1804* Ixodes ricinus* ticks were collected from 2010 to 2012. The ticks were collected from woodlands within the municipal Skodje (latitude: 62.507246680, longitude: 6.8334960937). At the collection site, there were grazing sheep from May to September, and roe deer were frequently observed. Individual ticks were placed in 1.5 mL Eppendorf tubes that were labelled with the date and geographic origin. No adult males were found when collecting ticks for this study. DNA isolation was performed using the DNeasy blood and tissue kit (Qiagen GmbH, Germany) as previously described [27].

The samples were analysed by qPCR to identify those samples containing* A. phagocytophilum*. A qPCR analysis was performed in a total reaction volume of 15 *μ*L using 7.5 *μ*L of TaqMan (no UNG) universal master mix (Applied Biosystems). The optimal reaction conditions contained 500 nM of each primer (primer 1; 5′-ATG GAA GGT AGT GTT GGT TAT GGT ATT-3′ and primer 2: 5′-TTG GTC TTG AAG CGC TCG TA-3′), 100 nM probe (5′-6FAM-TGG TGC CAG GGT TGA GCT TGA GAT TG-3′) [28], and 2 *μ*L of template DNA. The amplification was performed for* A. phagocytophilum* in a 7300 real-time PCR system (Applied Biosystems) with 1 cycle of denaturation (10 min, 95°C), followed by 45 cycles of denaturation (20 sec, 95°C) and annealing/extension (1 min, 60°C).

A multilocus amplification of all seven housekeeping genes was performed using nested PCR with primers as described by Huhn et al. [29]. The primer sequences are available through the MLST online database http://pubmlst.org/aphagocytophilum/. Both of the nested PCR amplification steps were performed in 15 *μ*L volumes, containing 7.5 *μ*L of Taq VWR master mix, 1.5 mM MgCl_2_, 0.75 *μ*L of each primer (10 *μ*M), 4.0 *μ*L of ddH_2_O, and 2 *μ*L of template DNA. The amplification was performed in a 2720 thermal cycler (Applied Biosystems, Carlsbad, USA) with one cycle of denaturation (10 min, 95°C), followed by a touchdown sequence of 12 cycles of denaturation (30 sec, 95°C), an annealing step (30 sec, decreasing temperature in 0.5°C increments from 59°C to 53°C), and extension (1 min, 72°C) and subsequent amplification by 28 cycles of denaturation (30 sec, 95°C), annealing (30 sec, 53°C), and extension (1 min, 72°C) and a final cycle of extension (10 min, 72°C).

To confirm the amplicon size and the approximate concentration of the amplified product, gel electrophoresis was performed for each amplified fragment. Gel electrophoresis was performed with a 2% Tris-acetate EDTA buffer (TBE) gel and prestained with GelRed (Affymetrix, Santa Clara, US). After 45 min of migration, the amplified fragments were visualised on the gel using UV light.

The PCR products were sent to Eurofins MWG (Germany) for custom DNA sequencing.

Statistical calculations of the qPCR results were performed using IBM SPSS statistics 20 (SPSS Inc., Chicago, USA). The chi-square test (*p* = 0.01) was used to examine the differences in the infection rate in the nymphal and adult* I. ricinus* ticks.

The MUSCLE algorithm was used to align and concatenate the sequences in the software MEGA 5.1 [30] and a bioinformatic analysis was performed. A phylogeny analysis was performed using the neighbour-joining method (boot-strapped 500 iterations) for concatenated DNA sequences. The nonredundant database (NRDB) written by Warren Gish, Washington University, was used to compare the allele sequences and sequences types (STs) and to identify identical sequences (available from http://pubmlst.org/analysis/) and known alleles; the STs were retrieved from the* A. phagocytophilum* MLST database (http://pubmlst.org/aphagocytophilum/). Pairwise genetic similarity was calculated based on the pairwise distances for individual isolates using the Kimura 2-parameter model. The modified Nei-Gojobori method (Jukes-Cantor) was used to calculate the average nonsynonymous substitutions and synonymous substitutions (dN/dS), and Tajima's Test of Neutrality was used to calculate the number of polymorphic sites (PS) and the nucleotide diversity (*π*). To compare the relationship between Norwegian* A. phagocytophilum* strains and* A. phagocytophilum* strains from the MLST database, a goeBURST full minimum spanning tree was generated using PHYLOViZ 1.0 software [31].

## 3. Results

The total number of collected ticks each year was 600 in 2010 (529 nymphs and 71 adult females), 603 in 2011 (538 nymphs and 65 adult females), and 601 in 2012 (540 nymphs and 61 adult females). The prevalence of* A. phagocytophilum* was demonstrated by qPCR analysis. A total of 56 samples were found to be* A. phagocytophilum* positive. The results indicate that the overall infection rate was 2.83%, 3.32%, and 3.16% in 2010, 2011, and 2012, respectively. Statistical analysis confirmed that there was no significant difference (*p* = 0.01) in the overall infection rate in 2010, 2011, or 2012.

To study the prevalence of* A. phagocytophilum* during different life stages, the results were divided into nymphs and adult females. The infection rate in adult females was approximately the same in 2010, 2011, and 2012 (Table 1). The statistical analysis indicated that the infection rates were significantly higher (*p* = 0.01) in adult females than in nymphs in 2011, but no statistically significant differences (*p* = 0.01) were observed in 2010 or 2012.

To describe the strain diversity, 30 samples were analysed with MLST (Table 2). All of the samples from each year of collection were submitted to amplification of the seven loci in the multilocus sequencing scheme, but not all of the samples amplified across all seven loci. In total, 30 samples amplified across all seven loci. An analysis of the sequences identified the sequence types and alleles. Of these sequences, 2 were adult females and 28 were nymphs. The 30 analysed strains were separated into 19 STs.

**Figure 1:**
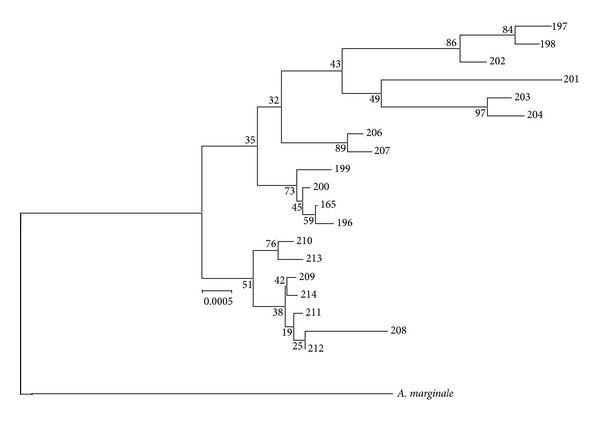
Neighbor-joining tree (boot strapped 500 iterations) with concatenated* A. phagocytophilum* sequences. The tree is rooted with sequences from* A. marginale*. All strains are numbered by their sequence type as described in Table 2.

The concatenate sequences were used to construct a neighbour-joining tree (bootstrapped 500 iterations) to demonstrate phylogenetic relationships (Figure 1). The tree was rooted using sequences from* Anaplasma marginale* from the NCBI database (http://www.ncbi.nlm.nih.gov/).

The calculation of the statistical parameters (Table 3) indicated that all of the loci have nucleotide diversity (*π*) < 0.01 and that most of the point mutations are synonymous (dN/dS). The percent of polymorphic sites (PS (%)) ranges from 1.0 to 2.80% (between 1 and 2.8 SNPs per 100 bp) given a selection of allele variants within each locus.

**Figure 2:**
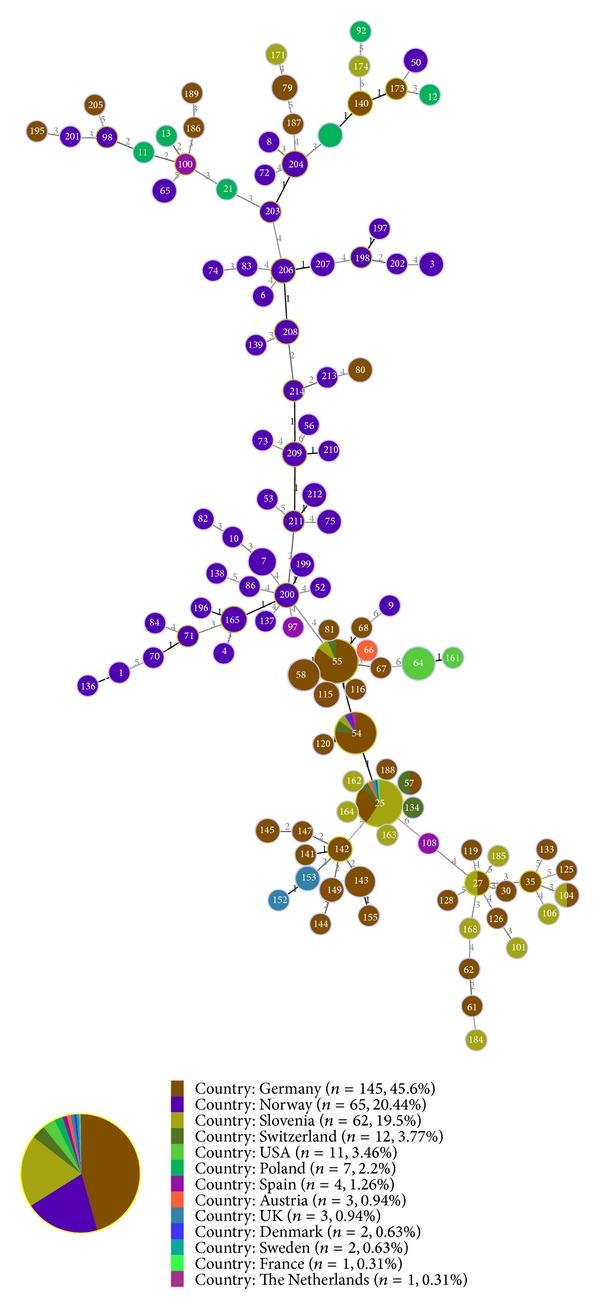
goeBURST full minimum spanning tree of STs from the* A. phagocytophilum* MLST database.

A goeBURST analysis (Figure 2) was conducted to compare the strains from* I. ricinus* ticks that were collected in northwest Norway to the strains in the MLST online database. The database contains sequence information on 317 strains from 13 different countries. These strains originate from a variety of reservoir hosts and some vectors.

The strains that were included in this study are a part of clonal complexes (CC) 9, 10, and 11, in addition to the singleton 201 (Table 4). The majority of the strains in the MLST database are from Germany, and most of these strains belong to CC1. The other Norwegian strains in the MLST database mainly originate from sheep (*Ovis aries*) that were diagnosed with TBF and are defined as singletons.

The majority of the strains in the MLST database are from central Europe (Germany and Slovenia), and these strains cluster together in one part of the goeBURST full minimum spanning tree (Figure 2). The Norwegian strains constitute the middle part of the goeBURST tree but with less concentration and longer branches than the central European strains.

## 4. Discussion

The tick-borne pathogen* A. phagocytophilum* constitutes an infection risk for livestock grazing in tick-infested areas. Although human infection is not widespread in Norway, TBF affects large quantities of the sheep in the county Møre og Romsdal [4]. In this study, the prevalence of the tick-borne pathogen* A. phagocytophilum* was determined in 1804* I. ricinus* ticks that were collected over a three-year period from 2010 to 2012. The overall infection rate was 2.83%, 3.32%, and 3.16% in 2010, 2011, and 2012, respectively [4]. Statistically, there was a significantly greater prevalence of* A. phagocytophilum* in adult females in 2011, but there were no significant differences in the infection rate between nymphs and adult females in 2010 or 2012. Generally, 2 to 3 of 100 ticks were infected by the pathogen, indicating that those areas with high tick density and an increased probability of multiple tick bites present a higher risk of infection.* A. phagocytophilum* can maintain itself without the presence of* I. ricinus* as vector, indicating that* I. ricinus* ticks may not be an important part of the natural cycle of* A. phagocytophilum* [32].

To characterise the* A. phagocytophilum* strains from the collected* I. ricinus* ticks, 30 positive samples were further analysed with multilocus sequence typing (MLST). The genotyping of 30 strains resulted in the classification of 19 different sequences types (ST), none of which is found in the MLST database. All STs have alleles that have been previously identified and submitted to the database but have combination of alleles that are new to the database. The strains display a number of polymorphic sites that create genetic diversity amongst the strains that were isolated from ticks. Interestingly, the genetic differences among the strains isolated from adult females (mean 0.002) are fewer than those among the strains isolated from nymphs (mean 0.018). The nucleotide diversity for the loci* dnaN*,* fumC*,* glyA*,* mdh*,* pheS*, and* sucA* is significantly higher in the strains isolated from nymphs than in the strains isolated from adult females. Nucleotide diversity is a reflection of the mutation rate per nucleotide site per host generation [33].* I. ricinus* ticks have a three-host cycle and are usually linked to different hosts during different life stages. Even though a number of potential hosts are available in the fauna, only a few selected species are selected as hosts [34]. Nymphs would have acquired their* A. phagocytophilum* bacterium while feeding on their first host as larvae. A study in Germany revealed that 97.9% of ticks feeding on rodents were larvae, and even with a large number of host seeking nymphs available the nymphs did not choose the same host as larvae [35]. Larvae do not seem to be suitable vectors for all* A. phagocytophilum* strains such as the field hole variant [36]. Host-specific vectors seem to keep* A. phagocytophilum* in certain niche cycles and help preserve* A. phagocytophilum* infection in nature [19, 36, 37]. The* A. phagocytophilum* strains may therefore originate from different host species in nymphs and adult females, indicating that* A. phagocytophilum* strains may be influenced by different evolutionary rates depending on the host-vector interaction to which the strain is subjected.* A. phagocytophilum* strains are mainly transmitted by* Ixodes* ticks, and vector competence has been demonstrated for the European ticks* I. ricinus* and* I. scapularis* and the American ticks* I. scapularis*,* I. pacificus*, and* I. spinipalpis*. Transovarial transmission (TT) has not been demonstrated for any of the Ixodes ticks but has been demonstrated in the one host tick* Dermacentor albipictus*. This distinct ecological niche demonstrates the ability of* A. phagocytophilum* to adapt [37].

The delineation of* A. phagocytophilum* has mainly been conducted based on single locus sequencing and clustering analysis. Studies based on the 16S rDNA sequences have begun to identify host-specific strain genotypes among the American strains, but the European strains do not seem to display the same characteristics [17, 38–40]. Compared to other housekeeping loci, such as* mps4*,* mps2*,* groEL,* and* ankA*, the 16S rRNA gene is too conserved to provide a high resolution phylogenetic analysis [12], but a phylogenetic analysis of* A. phagocytophilum* based on multiple, more heterogenic loci has the potential to delineate strains based on their ecotype and geographic origin [11, 16, 22, 36, 40, 41]. By combining the single-locus characteristic from more than one locus, the clustering patterns can be assigned to more than one ecological niche, providing a more detailed understanding of the strain diversity and genetic traits. This combination has been performed to demonstrate host-specific clustering based on the phylogeny of the ankA locus combined with characteristics from the 16S rRNA and groEL loci. The sequencing of multiple loci has demonstrated that it is necessary to determine the host-specific clusters [23, 36, 42]. The MLST scheme consists of seven housekeeping loci that separate genotypes based on nucleotide diversity within a concatenated fragment of 2877 bp, providing a high-resolution genotype, and the results are comparative worldwide. This method was recently developed, and the first study utilising MLST for population structure analysis was published in April 2014. Huhn et al. [29] reported that multiple strains among the samples were a limitation for their study [29]. Although the study of Huhn et al. included a range of animals and humans, this study included only ticks, and multiple strains did not seem to affect the sequencing. Comparing all of the concatenated sequences from northwest Norway to those in the MLST database, these sequences mainly cluster together with other concatenated sequences from Norway (Figure 2). The strains from this study cluster in CC 9, 10, and 11, indicating that these strains display clonal characteristics. The central European strains mainly cluster in CC1 and the database contains mostly strains that originate from humans or ruminants. The most distant concatenated sequences are those originating from the USA. The strains from the USA have a level 6 linkage in goeBURST, indicating that there are differences in 6 out of 7 loci. This result is consistent with previous studies that indicate that* A. phagocytophilum* strains develop differently in the USA than in Europe [29, 36].

The evaluation of polymorphic sites shows that there are more synonymous than nonsynonymous substitutions (dN/dS). The number of SNPs does not seem to affect the function of the genes and would mainly be spontaneous mutations as a result of mutagenic factors that are linked to a geographic location or specific environment. SNPs can be used to track generations [43]. A number of different SNPs have been identified in this MLST, but which of the identified SNPs are informative remains to be determined.

This study demonstrated the prevalence of* A. phagocytophilum* in* I. ricinus* ticks and applied a molecular genotyping tool, MLST, to study the strain diversity. The number of variants that were identified among the isolates demonstrates the highly discriminatory power of the MLST method and provides information about the genetic diversity among* A. phagocytophilum* strains from northwest Norway. Compared to the strains that have been isolated worldwide, the strains from northwest Norway cluster together with other Norwegian strains and constitute CC 9, 10, and 11. The genetic characteristics are slightly different from those of the central European strains, indicating that geographic origin may play a role in the evolution of* A. phagocytophilum* strains.

## Figures and Tables

**Table 1:** Statistical analysis of the infection rates in nymphs and adult female ticks.

*A. phagocytophilum* infection rates in 2010, 2011, and 2012						
	Not infected	Infected	Total	Chi-Square	*P* value	*p *
2010						
Nymph	514	15	529	0,000	1.000	0,01
Adult	69	2	71			
Total	**583**	**17**	**600**			

2011						
Nymph	521	17	538	0,420	0.711	0,01
Adult	62	3	65			
Total	**583**	**20**	**603**			

2012						
Nymph	523	17	540	0,003	1.000	0,01
Adult	59	2	61			
Total	**582**	**19**	**601**			

**Table 2:** The allele variants from each gene and the sequence types (ST) of *A. phagocytophilum* strains originating from Norwegian *Ixodes ricinus* ticks. The allele variants were identified by the nonredundant database, and STs were assigned.

Year	pheS	glyA	fumC	mdh	sucA	dnaN	atpA	ST
2011	27	6	6	3	5	2	1	165
2012	27	6	6	3	5	2	1	165
2010	27	6	6	3	5	2	1	165
2010	27	6	6	3	5	2	19	196
2011	43	1	7	3	2	2	19	197
2012	43	1	7	4	2	2	19	198
2010	27	6	6	3	88	53	1	199
2011	27	6	6	3	88	2	1	200
2011	27	6	6	3	88	2	1	200
2012	27	6	6	3	88	53	1	199
2010	21	2	13	8	11	2	1	201
2011	43	1	7	4	4	2	1	202
2011	23	9	7	4	11	2	2	203
2010	23	9	7	3	11	2	2	204
2010	23	9	7	3	11	2	2	204
2012	23	9	7	3	11	2	2	204
2010	27	6	13	4	47	2	2	206
2011	27	6	13	4	47	2	2	206
2012	27	6	13	4	47	2	19	207
2012	27	6	13	4	47	42	2	208
2012	27	6	29	4	88	42	1	209
2012	27	6	29	3	88	42	1	210
2011	27	6	6	4	88	42	1	211
2011	27	6	6	4	88	42	2	212
2011	27	6	6	4	88	42	2	212
2012	27	6	29	3	5	42	2	213
2010	27	6	29	4	88	42	2	214
2012	27	6	13	4	47	2	19	207
2010	27	6	13	4	47	42	2	208
2012	27	6	29	4	88	42	1	209

**Table 3:** Characteristics of each housekeeping gene.

Gene	Size (bp)	Average G+C content %	PS (%)^a^	*π* ^ b^	dN/dS^c^
*pheS *	438	56.2	2.51	0.0067	0.024
*glyA *	387	54.2	1.55	0.0026	0.008
*fumC *	411	52.2	2.68	0.0089	0.021
*mdh *	387	51.8	1.03	0.0042	0.015
*sucA *	429	57.7	2.80	0.0078	0.019
*dnaA *	405	51.4	2.22	0.0095	0.031
*atpA *	420	58.6	1.67	0.0037	0.007

^a^Percent polymorphic sites (PS (%)).

^b^Nucleotide diversity (*π*).

^c^Average nonsynonymous substitutions versus synonymous substitutions (dN/dS).

**Table 4:** Summary of the STs from this study and their respective clonal complex.

STs	Clonal complex
165, 196, 199, 200, 206, 207, 208, 209, 210, 211, 212, 213, 214	9
203, 204	10
197, 198, 202	11
201	None
